# Microtubule plus-end tracking proteins in neuronal development

**DOI:** 10.1007/s00018-016-2168-3

**Published:** 2016-03-11

**Authors:** Dieudonnée van de Willige, Casper C. Hoogenraad, Anna Akhmanova

**Affiliations:** Cell Biology, Faculty of Science, Utrecht University, Padualaan 8, 3584 CH Utrecht, The Netherlands

**Keywords:** Neuron, Development, Polarity, Cytoskeleton, Microtubule, Plus-end tracking proteins, EB, CLIP, CLASP

## Abstract

Regulation of the microtubule cytoskeleton is of pivotal importance for neuronal development and function. One such regulatory mechanism centers on microtubule plus-end tracking proteins (+TIPs): structurally and functionally diverse regulatory factors, which can form complex macromolecular assemblies at the growing microtubule plus-ends. +TIPs modulate important properties of microtubules including their dynamics and their ability to control cell polarity, membrane transport and signaling. Several neurodevelopmental and neurodegenerative diseases are associated with mutations in +TIPs or with misregulation of these proteins. In this review, we focus on the role and regulation of +TIPs in neuronal development and associated disorders.

## Introduction

Microtubules (MTs) are one of the major types of filaments that constitute the eukaryotic cytoskeleton. Over the years, MTs have emerged as key players in cellular processes such as vesicle and organelle transport, DNA segregation during mitosis, cell migration and maintenance of cell polarity. Neurons are among the most complex and polarized cells, whose distinct morphology allows them to establish intercellular connections and propagate chemical and electrical signals across the nervous system. Mature neurons typically extend multiple processes, one of which (the axon) serves as a transmitter whereas others (the dendrites) act as receivers of input from other neurons. MTs are important for numerous functions in nerve cells (reviewed in [1–3]), such as long-range transport of cargo and neuron-specific processes like growth cone guidance. Indeed, MTs are indispensable for neurodevelopment, and many neurological diseases stem from defects in the MT cytoskeleton or its regulation.

Evidence for the existence of MTs was first obtained from electron microscopy (EM) data in the 1950s (reviewed in [4]). Prior to the development of EM, fibrillar structures had already been described as part of the mitotic spindle and cytoplasm. However, interpretative differences and technical limitations of early microscopy made it challenging to identify unity among observations [4]. It therefore was not until 1963 that MTs were acknowledged as distinct structures and named by Slautterback, Ledbetter and Porter [5, 6]. Today, we know that MTs are hollow tubes with a diameter of approximately 25 nm. MTs are typically assembled from 13 laterally associating protofilaments, which in turn consist of α, β-tubulin dimers aligned in a head-to-tail fashion. As a consequence of tubulin dimer polarity, MTs possess polarity throughout, which results in distinct ends of the polymer: the minus- and the plus-end, exposing α- and β-tubulin, respectively. MTs alternate between rapid phases of growth and shrinkage, a behavior termed ‘dynamic instability’ [7]. A transition from shrinkage to growth is called a rescue, whereas the opposite transition is referred to as a catastrophe.

Dynamic instability allows MTs to be swiftly remodeled in response to environmental cues. The MT cytoskeleton is suitable for rapidly sensing and responding to changes in the intracellular environment. To this end, the MT cytoskeleton acts in concert with a large number of proteins (MAPs for MT-associated proteins) that either influence MTs themselves or relay signals from the MT cytoskeleton to other parts of the cell. MAPs are known to regulate MT behavior such as stability, assembly, bundling and targeting by associating with specific parts of the MT lattice or by interacting with the soluble tubulin pool. Well-characterized neuronal MAPs include MAP2 and tau, which maintain a polarized, mutually exclusive distribution and decorate MT bundles in dendrites and axons, respectively. Both proteins stabilize MTs and are able to induce MT bundling (reviewed in [8]). Abnormal phosphorylation of tau triggers its dissociation from MTs and causes tau to aggregate, resulting in the formation of potentially toxic tau deposits (neurofibrillary tangles) found in the brains of patients suffering from Alzheimer’s disease (AD) and other tauopathies. This process is accompanied by degradation of the axonal MT cytoskeleton, suggesting a model in which dissociation of tau results in MT instability. It should be noted, however, that the precise hierarchy of events during the onset of AD remains unclear. Additional roles for tau are still emerging and may shed new light on the biology of tauopathies (reviewed in [9]). Among these is the regulation of the subcellular distribution of MAPs that specifically bind to the growing MT plus-end [10], the subclass of MAPs that this review will focus on.

MT dynamics are most pronounced at the plus-end. Although growth events have been observed at the MT minus-end [11], in cells minus-ends are often anchored or stabilized, restricting their dynamic behavior [12]. At the growing plus-end, freshly polymerized MT stretches contain GTP-loaded β-tubulin as opposed to the GDP-bound subunits present in the MT lattice, resulting in a so-called GTP cap. Moreover, the structures of polymerizing and depolymerizing MT plus-ends are different [13]. The unique chemical environment of the polymerizing MT plus-end grants it its own interactome within the realm of MAPs, consisting of MT plus-end tracking proteins (+TIPs; reviewed in [14–16]). +TIPs display a large structural and functional variation between individual proteins. However, a common theme sets them apart from other MAPs: +TIPs associate with the polymerizing MT plus-end, where they act as powerful regulators of MT dynamics and MT interactions with other structures.

In this review, we use the neuronal MT cytoskeleton to illustrate the role of +TIPs in the development of one of the most polarized and complex cell types. Before discussing plus-end tracking mechanisms and highlighting the roles and regulation of various +TIPs in neurons, we touch upon the function of the MT cytoskeleton in the developing and mature nervous system. Moreover, we highlight the role of +TIPs in neurodegenerative and neurodevelopmental diseases. We conclude this review with an outlook on the future of neuronal +TIP research and briefly discuss the drug target potential of these pivotal proteins.

## Microtubules in neurons

Neurons are derived from progenitor cells located in the ventricular zone deep inside the brain, necessitating young neurons to migrate large distances into remote regions. During their journey, neurons undergo dramatic changes in morphology and establish complex polarity. Even mature neurons must remain plastic as connections between neurons, synapses, are continuously rewired in response to stimuli. This intricate development relies heavily on both the MT and the actin cytoskeleton, on their crosstalk and on their accessory proteins. For a detailed analysis of the role of the cytoskeleton and in particular MTs during neuronal development, we refer the reader to a number of excellent reviews [1–3, 17]. Here, we briefly highlight some of the main events involving MTs during the maturation of multipolar neurons. It should be noted that the development of neurons extending a single process, unipolar neurons, is considerably different [18].

### Microtubules during neurite formation and axon outgrowth

**Fig. 1:**
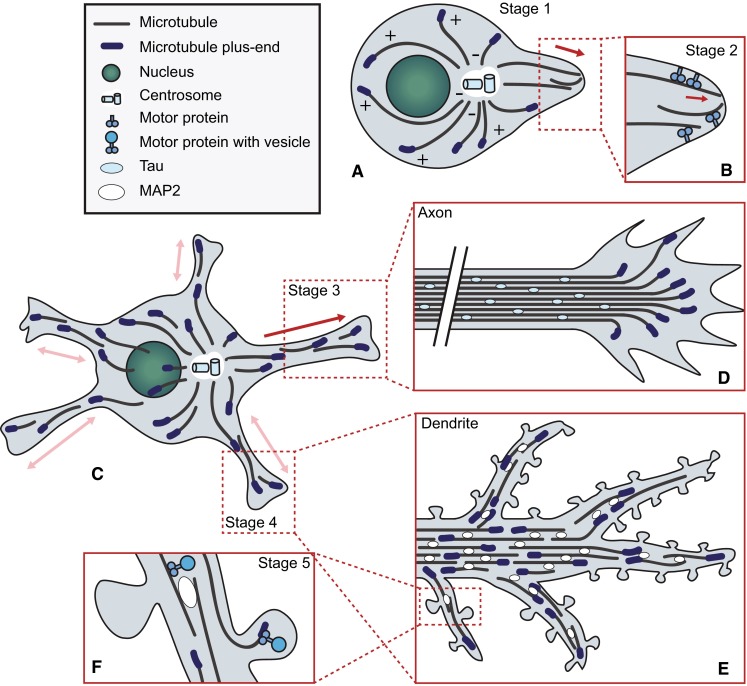
Microtubule organization and function during neurodevelopment. Cultured dissociated neurons start out as spherical, unpolarized cells with MTs oriented with their plus-ends towards the plasma membrane (**a**). Upon symmetry breaking, neurite extension is thought to be facilitated by motor proteins, which were proposed to push MTs and thus exert a force on the membrane to form protrusions (**b**). Young neurons possess multiple neurites and maintain a mainly plus-end out MT orientation (**c**). While remaining neurites cycle between phases of growth and shrinkage, one neurite rapidly extends to form the axon. In this neurite, MTs become stabilized and MT bundles are decorated with the axon-specific MAP tau, while MTs remain oriented plus-end out. The rate of advance and the directionality of axon outgrowth is controlled by the growth cone, a specialized structure at the tip of the axon that contains a dynamic array of MTs. Local stabilization of a MT in one of the filopodia of the growth cone prompts the growth cone to turn in that direction (**d**). Later in development, the remaining neurites differentiate into dendrites. Dendrites acquire unique antiparallel MT bundles decorated by MAP2, presumably contributing to selective cargo trafficking (**e**). The post-synapse is present at the tips of dendritic spines. Targeting of dynamic MTs to spines triggers morphological changes and alters synaptic strength, possibly by allowing the delivery of specific cargo to the spine or activating signaling processes (**f**)

Neurons start out as spherical, unpolarized cells with a MT organization similar to that commonly found in mammalian cells (Fig. 1a). In young neurons, MTs mainly nucleate from the centrosome, with MT minus-ends pointing inward and plus-ends oriented towards the cell periphery [19]. Upon differentiation, neurons undergo symmetry breaking. During this event, the neuron extends multiple processes that start as small buds on the membrane and elongate to form thin protrusions [20]. These early protrusions, termed neurites, mature into axons and dendrites when the neuron polarizes. It has been proposed that neurite formation is powered, at least in part, by MT sliding. One model suggests that MAP2c (microtubule-associated protein 2c) induces stable MT bundles, which translocate to the membrane where they exert a dynein-dependent force to trigger protrusion formation [21]. Another model proposes that the motor protein kinesin-1 powers the displacement of MTs along other MTs, exerting a mechanical force on the membrane which results in neurite extension [22] (Fig. 1b).

Shortly after neurite extension, the axon is formed (Fig. 1c). This event is preceded by local stabilization of MTs in the pre-axonal neurite [23]. As the newly formed axon starts to elongate, it relies on stable MT tracks for the transport of proteins and organelles necessary for the formation of new axonal segments [24]. The direction of axon outgrowth is determined by the growth cone, which probes the extracellular environment to allow non-random establishment of synaptic connections. Amidst the actin, which drives invasion of the extracellular matrix (reviewed in [25]), an array of MTs controls the direction in which the growth cone advances (Fig. 1d; [26]). The MT array assumes a looped conformation in pausing growth cones [27], while active growth cones maintain a dynamic MT array. These MTs probe the growth cone cortex and respond to guidance signals by being stabilized or destabilized, prompting the growth cone to turn towards or away from the guidance cue, respectively [24, 28]. As the axon matures further, it branches to allow higher interconnectivity. Branch formation is accomplished by splaying of tau-decorated MT bundles at branching sites. Here, dynamic MTs invade actin-rich areas close to the membrane to form a collateral branch [29]. Thus, while dynamic MTs play only a minor role in neurite outgrowth, they are crucial for axon polarization, pathfinding and branching.

### Microtubules during dendrite maturation

Dendritic differentiation occurs later in development than axogenesis and prompts dramatic changes to the soon-to-be dendritic MT infrastructure. Prior to dendritogenesis, mammalian precursor neurites cycle between phases of growth and shrinkage and maintain ~80 % plus-end out^1^ MT directionality [30]. Perhaps the most striking feature of the dendritic MT cytoskeleton is the appearance of MTs with their minus-ends oriented towards the dendritic tips [31, 32] (Fig. 1e). However, it should be noted that the ratio between minus- and plus-end out MT orientations changes per neuron type and even differs between regions of the same dendrite. In invertebrates, as much as ninety percent of dendritic MTs are oriented minus-end out [33, 34]. Differences in MT orientations between axon and dendrites likely contribute to proper targeting of specific cargo by enabling transport by specific motor proteins. While dendrites branch more than axons, the behavior of MTs during this process has been studied less extensively. Specialized Golgi compartments called Golgi outposts were identified as potential sites of MT nucleation in centrosome-free *Drosophila* neurons [35], and were shown to localize to dendritic branch points [36]. Since axon branching depends on dynamic MTs arising from splayed bundles, it seems likely that dynamic MTs nucleated at Golgi outposts fulfill a similar role in dendritic branching [35].

### Microtubules and synapses

The formation of synapses, connections between the axon of one neuron and the dendrite of another, is the final and ongoing step in neuronal maturation. Excitatory synapses are formed on dendritic spines, which are actin-rich protrusions on the dendrite that scaffold the post-synaptic density. The absence of MAP2-positive MTs in dendritic spines raised the belief that the MT cytoskeleton remained confined to the dendritic shaft [37]. However, visualization of the behavior of MTs and MT plus-ends revealed that dynamic MTs transiently invade dendritic spines by polymerizing from proximal sites [38–41]. MT-depolymerizing drugs such as nocodazole markedly reduce the number of spines, while the number of dendrites remains unaffected [38]. Nocodazole also blocks the spine-inducing effect of the growth factor BDNF (brain-derived neurotrophic factor), while the MT stabilizing drug taxol enhances BDNF-induced spine formation [40]. Finally, depletion of end-binding protein 3 (EB3), a key scaffolding factor at the MT plus-end and regulator of MT dynamics discussed below, reduces the amount of spines [40]. These studies imply dynamic MTs as modulators of neuronal plasticity. +TIPs involved in synaptic remodeling may rely on dynamic MT plus-ends as a means of reaching spines targeted for remodeling [38]. Other candidate regulatory mechanisms involve the facilitation of cargo delivery to the postsynaptic terminal [1] (Fig. 1f), though it remains to be elucidated exactly which events are at play.

## Microtubule plus-end tracking proteins in neurons

MT behavior during neurodevelopment has been catalogued extensively. However, research has only just begun to uncover which factors control MT dynamics and how MTs are able to relay intricate signals in neurons. Many of the cellular processes and the molecular mechanisms that underlie them remain unknown to date.

Since +TIPs have emerged as potent MT regulators, they are excellent candidates to control MTs and relay their signals during neuronal development and homeostasis. Indeed, numerous +TIPs have already been linked to neurodevelopmental functions (Table 1). New +TIPs are still discovered on a regular basis, and it is likely that additional roles for +TIPs in neurons will be uncovered in the future. Current knowledge suggests that, based on their mode of association with MTs, +TIPs can be divided into three categories: end-binding proteins (EBs), EB-dependent +TIPs and EB-independent +TIPs. It should be noted that even in the case of EB-independent +TIPs, there is crosstalk between these +TIPs and EBs albeit indirectly. Therefore, none of these categories can be regarded as fully independent.

**Table 1:** Overview of +TIPs with confirmed functions and/or human disease significance in the nervous system

+TIP (common aliases)	Mode(s) of MT plus-end association	Reported involvement in neurodevelopment	Human neurological disease association(s)	References
Amer2 (FAM123A)	SxIP motif	Neuronal migration		[188]
APC (DP2.5)	Autonomous; SxIP motif; kinesin-dependent	Neuronal migration; neurite outgrowth; axon specification; axon outgrowth; axon branching; growth cone steering; synaptic maturation	Autism; brain tumor-polyposis syndrome 2	[189–199]
APC2 (APCL)	SxIP motif	Neuronal migration; axon branching; growth cone steering	Sotos syndrome	[200–202]
CDK5RAP2 (Cep215)	SxIP motif	Neural progenitor cell division	Autosomal recessive primary microcephaly (ARPM)	[203–206]
CEP104 (KIAA0562)	SxIP motif		Joubert syndrome	[207]
ch-TOG (CKAP5)	Autonomous	Axon outgrowth		[128, 129]
CLASP1/2 (1: hOrbit1, KIAA0622; 2: hOrbit2, KIAA0627)	SxIP motif	Axon outgrowth; growth cone steering; dendritic branching; synaptic maintenance; synaptic activity		[90, 91, 93–96]
CLIP-115/170 (−115: CLIP2, WBSCR3, WBSCR4, KIAA0291; −170: CLIP1, CYLN1, restin)	CAP-Gly domain	Axon formation; axon outgrowth; growth cone dynamics; dendritic outgrowth; dendritic branching	Williams syndrome (CLIP-115); autosomal recessive intellectual disability (CLIP-170)	[82, 83, 85, 208]
CTTNBP2 (CORTBP2, C7Orf8, KIAA1758)	SxIP motif	Dendritic branching; dendritic spine formation; dendritic spine maintenance; synaptic signaling	Autism	[209–213]
DDA3 (PSRC1, FP3214)	SxIP motif	Neurite outgrowth; axon formation		[214]
EB1–3 (MAPRE1–3)	Autonomous	Neurite outgrowth; axon formation; dendritic branching; AIS maintenance; +TIP scaffolding^a^		[64, 65, 67, 69, 215, 216]
FILIP1 (KIAA1275)	SxIP motif	Neuronal migration		[217]
iASPP (PPP1R13L, NKIP1, RAI)	SxIP motif	Neuronal fate after injury	Glioma; stroke	[218–221]
KIF2C (MCAK, kinesin-13)	SxIP motif; Plus-end directed motor activity		Glioma	[222]
KIF11 (Eg5, TRIP5, KNSL1)	SxIP motif	Neuronal migration; neurite outgrowth; axon outgrowth; axon branching; growth cone steering; dendritic outgrowth; cell surface receptor transport	Microcephaly with or without chorioretinopathy, lymphoedema, or mental retardation (MCLMR); glioma	[223–231]
LIS1 (PAFAH1B1)	Via CLIP-170	Neural progenitor cell division; neuronal migration; neurite outgrowth; axon outgrowth; dendritic outgrowth; dendritic branching; synapse formation; synaptic activity; dynein-based transport	Lissencephaly; subcortical band heterotopia (SBH)	[232–247]
MACF1 (ACF7, macrophin-1, ABP620, trabeculin-alpha, KIAA1251)	SxIP motif	Neuronal migration; axon outgrowth; axon branching; dendritic branching; dendritic spine maturation	Spectraplakinopathy type 1	[63, 99, 101, 144, 248, 249]
MACF2 (dystonin, BPAG1, CATX15, trabeculin-beta, KIAA0728)	SxIP motif	Axonal transport; maintenance of axonal cytoskeleton integrity; maintenance of Golgi integrity; ER stress level regulation; autophagy	Dystonia; hereditary sensory autonomic neuropathy	[104–109]
Neuron navigators 1–3 (NAV1/2/3; Steerin-1/2/3; Unc53H1/2/3; −1: POMFIL3, KIAA1151, KIAA1213; −2: HELAD1, RAINB1, POMFIL2, KIAA1419; −3: POMFIL1, KIAA0938)	SxIP motif	Neuronal migration; neurite outgrowth; axon outgrowth	Neuroblastoma (NAV3)	[135, 250–258]
P140Cap (SNIP, SRCIN1, KIAA1684)	SxIP motif	Dendritic spine formation; dendritic spine maintenance; synaptic vesicle secretion		[38, 113, 115, 116, 259, 260]
p150glued (dynactin subunit 1, DCTN1, p135, DAP-150)	CAP-Gly domain	Dynein-based transport	Perry syndrome; hereditary motor neuropathy 7B (HMN7B); amyotrophic lateral sclerosis (ALS)	[157, 159, 162, 163, 261, 262]
SLAIN1/2 (−1: C13orf32; −2: KIAA1458)	SxIP motif	Axon outgrowth		[128]
STIM1 (GOK)	SxIP motif	Neural differentiation; growth cone steering; SOCE	Amyotrophic lateral sclerosis (ALS); neurogenic muscular atrophy; Huntington’s disease; neuroblastoma; brain damage after insult or injury	[118, 119, 263–269]
Syntabulin (Golsyn, KIAA1472)	SxIP motif	Axonal transport; synaptic plasticity; mitochondria trafficking		[270–273]
TACC3 (ERIC1)	Unclear	Neural progenitor cell division; neuronal differentiation; axon outgrowth		[274–277]
TRIO (ARHGEF23)	SxIP motif	Neuronal migration; neurite outgrowth; axon outgrowth; growth cone steering		[258, 278–285]
TTBK1 (KIAA1855)	SxIP motif		Alzheimer’s disease; Amyotrophic lateral sclerosis (ALS); frontotemporal lobar degeneration (FTLD-TDP)	[176–178, 286]
TTBK2 (TTBK, KIAA0847)	SxIP motif	Ciliogenesis; GABA/osmolyte transport; neuronal migration	Spinocerebellar ataxia type 11; amyotrophic lateral sclerosis (ALS); frontotemporal lobar degeneration (FTLD-TDP)	[167, 169, 172, 174, 178]

^a^Note that due to their core function at the MT plus-end, it is difficult to separate EB roles in neurodevelopment from their scaffolding function

### End-binding proteins

EBs are at the core of the MT plus-end interactome. They are known to regulate MT behavior both autonomously and by providing a structural scaffold for other +TIPs [42–44]. EB plus-end tracking depends on an N-terminal calponin homology (CH) domain that grants MT affinity [45]. It has been shown that EB proteins associate with the MT plus-end by a CH-dependent nucleotide sensing mechanism [46, 47]. MT binding is regulated by a negatively charged C-terminal domain, which repels the negatively charged MT lattice and thereby contributes to specificity for the MT plus-end [48]. In addition, C-terminal coiled-coil and EB-homology domains mediate homo- and heterodimerization as well as interaction with other proteins including +TIPs [49, 50].

EBs mainly function as scaffolding proteins at the MT plus-end, where they form a hub for other +TIPs to associate with and thereby regulate local protein composition and MT dynamics. This is illustrated by the fact that EBs promote catastrophes when reconstituted with tubulin in biochemical preparations, while they reduce the number of catastrophes and promote continuous MT growth in cells. This suggests that EBs primarily act on other MT regulators in cells rather than autonomously [44]. Plus-end bound EBs rapidly exchange with the cytosolic pool, providing a rapidly remodeling platform for protein binding [43, 51]. Quantitative proteomic studies of non-neuronal cell lines have revealed that EBs are the most abundant plus-end binding proteins: the EB family outnumbers the second most abundant +TIPs by factors of approximately 7–30 in cultured fibroblasts [52, 53]. While technical limitations arising from sample heterogeneity have hampered large-scale quantitative proteomics studies in nerve cells [54], it is expected that EBs dominate neuronal MT plus-ends in a similar fashion as in cultured fibroblasts. The relatively high concentration of EBs compared to other +TIPs offers a simple explanation for how EB-decorated plus-ends are efficiently formed and maintained. Such a hub provides cells with an extra layer of control to regulate large numbers of +TIPs with minimal changes to the MT cytoskeleton itself, making it easier to retain MT integrity and reliant functions alongside.

In mammalian cells, the EB family is represented by three members (EB1, EB2 and EB3), which all bind to MT plus-ends but differ in their affinity for MT tips, phosphorylation and affinity for binding partners [44, 55–59]. EB1 and EB2 appear to be expressed ubiquitously, while EB3 is strongly expressed in muscle and brain tissue [60, 61]. During neurodevelopment, EB1 expression decreases while EB3 expression is upregulated [38]. Axon extension coincides with EB1 expression in neuroblastoma cells [62]. In *Drosophila*¸ depletion of EB1 impairs axon outgrowth and leads to the disorganization, but not loss, of MTs [63]. Other studies also point in the direction of a role for EB1 in axogenesis [64], and suggest a differential role of EB proteins in neurite formation. EB1 and EB3 have a positive role in neurite outgrowth, while EB2 has a negative effect [65], possibly because EB1 and EB3 have a higher affinity than EB2 for MT-stabilizing partners [56, 66]. EB3 has been specifically implicated in neuritogenesis in the context of actin-MT interactions [67], suggesting that the mechanisms underlying the importance of EBs for controlling neuronal morphogenesis can be quite complex.

Apart from scaffolding other +TIPs, EBs may also aid the capture of MT plus-ends for regulatory purposes. For example, MTs are proposed to rely on EB3 and Drebrin to enter actin-rich spines. Drebrin interacts with the growing MT plus-end via an unconventional interaction with EB3 [67], and is enriched in spines by binding to F-actin [68]. Drebrin localization becomes enhanced in spines upon NMDA (*N*-methyl-d-aspartate) receptor activity, whereupon Drebrin is believed to capture EB-decorated MT plus-ends near or in the spine neck and thereby guide dynamic MT entry into spines. Accordingly, Drebrin overexpression and increased amounts of F-actin upregulate the number of MT entries into spines [41].

Interestingly, another neuron-specific role for EB1/3 was reported that does not depend on plus-end tracking. EBs are enriched in the axon initial segment (AIS) of hippocampal neurons, where they contribute to AIS integrity and maintenance [69]. This possibly contributes to enhanced MT stability in the AIS [69], although the precise mechanism remains unclear.

### EB-dependent +TIPs: CAP-Gly proteins

Most known EB-dependent +TIPs can be divided in two categories, depending on their mode of association with EB proteins. The first category consists of a minority of +TIPs that contain an evolutionarily conserved cytoskeletal-associated protein glycine-rich (CAP-Gly) domain, which associates with EEY/F motifs in the C-terminus of EB proteins and tubulin [70–73].

An example of one such CAP-Gly domain-containing +TIP is p150glued: the largest out of eleven subunits of the dynactin complex. Dynactin is essential to nearly all functions of dynein, the most prominent minus-end directed motor protein [74]. Dynein plays a particularly important role in axons, wherein transport into the soma relies on minus-end directed transport due to the uniform plus-end out orientation of MTs. It is likely that multiple mechanisms contribute to dynein activation at different locations along the neuron [74]. At the MT plus-end specifically, one such model explains how retrograde transport is initiated when the dynamic MT plus-end loaded with p150glued and other dynein regulators encounters minus-end directed cargos ([75] and reviewed in [74]). While the role of dynactin in dynein plus-end targeting appears to differ between organisms [74], research suggests that dynactin accumulation at the MT plus-ends in axons of murine dorsal root ganglia (DRG) neurons contributes to long-range retrograde transport by recruiting dynein to vesicles [76]. It has been postulated that this function is exerted by a neuron-specific p150glued isoform, which reduces the frequency of catastrophes and thus increases MT stability [77].

Although p150glued binds to EBs directly, its affinity for MT plus-ends appears not to be very high. In cells, p150glued is assisted in targeting the plus-ends by another CAP-Gly domain containing protein, cytoplasmic linker protein of 170 kDa or CLIP-170 [76, 78]. Mammals also express a protein closely related to CLIP-170: the neuronally enriched CLIP-115 [79]. Both CLIPs are +TIPs, but differ by the structure of their C-termini. Only CLIP-170 contains zinc-binding domains and an EEY/F motif, which mediate the interactions with the CAP-Gly domain of p150glued and with the dynein regulator LIS1, as well as autoinhibition [78, 79]. Both CLIPs also promote MT rescue [80], although the underlying mechanism is still obscure, as it should involve depolymerizing MT ends or the MT lattice, where CLIPs are not enriched. Of note, CLIP plus-end tracking behavior is less prominent in neuronal compared to non-neuronal cells [81], which would be compatible with a function that is not directly related to growing MT tips. In addition to regulating dynactin recruitment, CLIPs are enriched in axonal growth cones, where they stabilize MTs protruding into the actin-rich leading edge [82]. CLIPs are therefore necessary for axon formation and outgrowth as MT stabilization in the growth cone precedes engorgement and consolidation. CLIP-170 is involved in dendrite morphogenesis by regulating crosstalk between the actin cytoskeleton and dynamic MTs [83]. Given the importance of CLIPs for different processes in cultured neurons, the phenotypes of CLIP-115 and CLIP-170 knock-out mice are rather mild, though CLIP-115 knock-out animals do display behavioral phenotypes [84, 85]. The loss of CLIP-190, the *Drosophila* homologue of CLIP-170, causes no strong phenotype either [81]. This suggests that the neuronal function of CLIPs might be redundant with that of other MT regulators.

### EB-dependent +TIPs: SxIP proteins

The largest subclass of EB-dependent +TIPs comprises proteins which utilize a short linear motif known as the SxIP motif (serine/threonine-any amino acid-isoleucine/leucine–proline) to bind the EB homology domain ([42] and reviewed in [14]). SxIP motifs are generally embedded in unstructured amino acid stretches enriched in proline, serine and basic residues, resulting in a positive charge [42]. Further computational analysis revealed that the nine amino acids surrounding the SxIP motif cannot contain acidic amino acids, and that at least one basic amino acid is present in the four amino acids preceding the motif [56]. Since discussing all currently identified SxIP +TIPs is beyond the scope of this review, we here focus on a selection of prominent examples to illustrate the broad range of neurodevelopmental functions of these +TIPs. All currently known neurodevelopmental functions are listed per +TIP in Table 1, along with their mode of association with the MT plus-end and the known neurological disease associations.

Among the most conserved SxIP proteins are CLASPs (cytoplasmic linker protein-associated proteins), the mammalian versions of which were discovered through their association with CLIPs [86]. Similar to CLIPs, there are two CLASP-encoding genes in mammals: *CLASP1*, which is expressed ubiquitously, and *CLASP2*, the products of which appear enriched in nervous tissue [86]. CLASPs utilize their SxIP motifs to bind EB1 and contain several additional TOG (tumor overexpressed gene) domains which can serve as tubulin-binding modules [87–89]. Different cell lines have revealed a function for CLASPs at the membrane, where they capture dynamic MT ends to promote MT rescue and pausing, and thus stabilize MTs [89, 90]. CLASP-mediated cortical MT stabilization is crucial to axon outgrowth and directionality and as such, CLASP was implicated in axon development in various organisms [90, 91]. Interestingly, CLASPs have affinity for both the MT plus-end and lattice, and differences in CLASP distribution and CLASP-MT associations inside the growth cone are able to direct axon growth status. CLASPs can be localized to the tips of growth cone filopodia, where they capture plus-ends of MTs to facilitate axon outgrowth. Conversely, in pausing growth cones, lattice-binding CLASP is present close to the end of the axon shaft to prevent MTs from protruding into the peripheral growth cone, thereby preventing outgrowth. The localization of CLASPs inside growth cones is regulated by kinases such as GSK3β and Abelson kinase [90, 92–94]. In addition to axon growth status, the direction of axon outgrowth is regulated by CLASP localization. The kinase-controlled, asymmetric distribution of CLASPs to the filopodia of a growth cone determines the sites of MT capture and thereby dictates the direction in which the axon advances [94, 95]. Additional functions for CLASPs include a role in synaptic functioning likely via global control of neuronal morphology [95] and maintenance of the *Xenopus* growth cone lamellipodium [91]. Furthermore, CLASP2 mediates MT capture at the postsynaptic membrane to promote transport of acetylcholine receptors to neuromuscular junctions [96].

Morphological changes during neurodevelopment are the result of complex interplay between different components of the cytoskeleton. This is in part facilitated by the Microtubule-Actin Crosslinking Factor proteins MACF1 (ACF7) and MACF2 (dystonin), known as spectraplakins. As spectraplakin nomenclature is complicated, we will here refer to MACF1/2. The reader is directed to Table 1 for a comprehensive list of alternative names for these proteins. Spectraplakins gain their name from membership of the spectrin family and their plakin repeats, which grant affinity for intermediate filaments [97]. Spectraplakins also contain CH domains to bind to actin, GAR (growth arrest-specific 2 protein-related region) domains to bind and stabilize MTs [98], and SxIP motifs to bind the MT plus-end via EBs. This places them at the heart of cytoskeletal crosstalk and renders them a popular subject for neurodevelopmental research. Indeed, homozygous MACF1 knockout mice are not viable, and mutant mice die from neuronal migration defects when MACF1 is depleted during development [99]. MACF1 is known to guide MTs along actin filaments and to mediate MT capture at actin-rich sites near the membrane [100], consistent with a role for MACF1 and its orthologs in growth cone MT organization and axon extension [101]. In case of the *Drosophila* MACF1 ortholog Shot, this function was shown to depend on its SxIP motifs and interaction with EB1 [63]. MACF1 has also been implicated in formation of growth cone filopodia, although for Shot this function does not rely on its actin- or MT-binding domains [101].

The second mammalian spectraplakin, MACF2 or dystonin, is best known for its role in the neurological disorder dystonia. MACF2 knockout mice develop dystonia and show repetitive muscle spasms [102], and mutations in MACF2 have been identified in patients with Hereditary Sensory Autonomic Neuropathy [103]. These pathologies are associated with the degeneration of sensory and autonomic nerves [102, 103]. Axons of MACF2 null mice degenerate as a result of MT fragmentation, which is believed to contribute to the dystonia phenotype independent of the neurofilament-binding functions of MACF2 [104]. MACF2 has also been implicated in retrograde axonal transport by interacting with p150glued [105]. This function may depend on MACF2’s EB-dependent association with the MT plus-end, as overexpression of peptides which competitively block EB–SxIP interactions inhibit retrograde transport of endosomes [106]. Another important factor in dystonia may be the neuron’s inability to regulate ER stress levels, Golgi integrity, MT acetylation and autophagy due to loss of a neuron-specific MACF2 isoform (BPAG1-a2) [107–109]. This particular isoform has an N-terminal trans-membrane domain and does not localize to MTs [110], although it does affect MTs near the centrosome via an association with MAP1B [108] and retains the C-terminal SxIP motifs. The same isoform was shown to partially rescue phenotypes in a *dystonia musculorum* mouse model [111]. On the other hand, another neuron-specific MACF2 splice variant (BPAG1n3) exclusively binds MTs and may be involved in sustaining axonal MT integrity [104], suggesting distinct functions for different MACF2 isoforms. The same holds true for the many splice variants of MACF1, and more research is needed to elucidate the contribution of spectraplakin plus-end tracking to their functions in both nerve cells and in other cell types.

+TIPs also play important roles in mature neurons, as neurons remain plastic throughout their lifespan and remodel synapses in response to both intra- and extracellular cues. One such +TIP with a function at the synapse is p140Cap, whose name is a combination of its molecular weight and ‘Cas-associated protein’ (Cap; Cas for Crk-associated substrate). p140Cap is regarded as a tumor suppressor protein due to its function as an inhibitor of Src kinase, which is involved in cell migration and growth [112]. In addition to its potential to associate with MT plus-ends via an SxIP/EB3-mediated interaction, p140Cap binds actin fibers and localizes to actin-rich dendritic spines of hippocampal neurons [38, 112]. p140Cap knockout mice display impaired learning and memory functions, and spine defects have been observed in the absence of p140Cap both in cultured primary neurons and in knockout mice [38, 113]. Synaptosomes prepared from *p140Cap−/−*- mice reveal hyperactivation and hyperphosphorylation of Src kinase and its substrate cortactin, respectively, as well as reduced RhoA activity [113]. Research suggests that p140Cap forms a synaptic complex with and increases the interaction between Src kinase and Citron-N, a protein known to scaffold the actin remodeling machinery, and as such controls spine morphology [113]. EB3 appears to function upstream of the spine remodeling process as synaptic phenotypes of EB3 knockdown mimic those of p140Cap knockdown, and can be rescued by simultaneous overexpression of p140Cap or Citron-N [38, 113]. Notably, while overexpressed p140Cap tracks MT plus-ends in neurons, this is rarely the case for endogenous p140Cap. p140Cap’s affinity for local binding partners in spines is likely sufficiently high to prevent cytoplasmic diffusion necessary for MT plus-end tracking behavior [38, 114]. In addition to its function at the postsynapse, p140Cap likely plays a role at the presynapse where it interacts with several proteins implicated in synaptic vesicle secretion [115, 116].

Another interesting SxIP-containing partner of EB1 is the Stromal interaction molecule 1 or STIM1 [117], a transmembrane ER protein which contains one SxIP motif and can thus link EB-decorated MT plus-ends to the ER membrane. STIM1 regulates store operated calcium entry (SOCE) in neurons and is necessary to resupply the ER with calcium by opening plasma membrane channels after calcium release during synaptic signaling [118, 119]. The function of the interaction of STIM1 with MT tips is not yet entirely clear. STIM1 participates in ER tubule extension by coupling growing MT plus-ends to the ER [117], and it is possible that such ER remodeling contributes to SOCE in certain cell types by bringing STIM1 in the vicinity of the plasma membrane. Interestingly, in HEK293T cells changes in MT dynamics affect STIM1’s association with calcium channels [120], suggesting that in some cell types MT dynamics are important for calcium signaling, possibly via the action of +TIPs. It remains to be verified, however, what the exact contribution of STIM1’s plus-end tracking behavior is to SOCE in neurons. Part of the answer may come from the function of STIM2, a STIM1 homolog with 63 % sequence identity but without conservation of the SxIP motif. STIM2 regulates SOCE instead of STIM1 in pyramidal neurons of the neocortex [121], suggesting that plus-end tracking is not required for STIM functioning during SOCE in neurons. However, remodeling of the ER was observed along MTs during SOCE [122]. As STIM1 was found to remodel the ER via its interaction with EB1 in HeLa cells [117], there is a possibility STIM1 may exert additional, potentially plus-end tracking-dependent functions in neurons. This is underlined by the finding that STIM1 participates in growth cone steering, and that this effect is only coupled to effects on SOCE for certain guidance cues [118].

To summarize, SxIP motif-containing proteins represent a large and heterogeneous group of EB-dependent +TIPs, which exert different functions at different stages of neurodevelopment. Although many roles have been identified (Table 1), it is not always clear what the contribution of plus-end tracking behavior is to each of these functions.

### Other +TIPs

While EB proteins are responsible for targeting a large variety of proteins to MT tips, several major classes of MT plus-end interacting factors target the growing MT plus-end via other mechanisms. These include the MT polymerase ch-TOG/XMAP215 and certain kinesin motor proteins, such as kinesin-4, -8 and -13 family members (see below). In addition, for some MT- or tubulin-binding proteins, such as doublecortin and stathmin, specific interaction with MT plus-ends was established or proposed based on in vitro reconstitution experiments [123, 124]. However, this was not demonstrated in cells, and is therefore not further discussed here.

ch-TOG/XMAP215 can track the growing plus-ends of MTs directly by recognizing the outmost MT tips [125, 126], or indirectly, via EB proteins. In mammalian cells, MT plus-end tracking of ch-TOG is facilitated by binding to SLAIN proteins, which themselves are EB-dependent, SxIP-containing +TIPs that have the ability to bind a number of other +TIPs [127]. Both SLAIN1/2 and ch-TOG are enriched in mammalian brain tissue, and promote MT growth by positioning the MT polymerase at the tip of the growing MT [128]. Like the majority of +TIPs implicated in maintaining MT cytoskeleton integrity, ch-TOG plays a role in axon outgrowth. Depletion of ch-TOG increases catastrophe rates in all subcellular compartments of rat hippocampal neurons, while reducing MT growth rates [128]. Axon outgrowth defects are apparent in young neurons depleted of ch-TOG, as well as in those overexpressing a dominant negative SLAIN construct that prevents ch-TOG from accumulating at the MT plus-end [128]. In *Xenopus*, the ch-TOG homolog XMAP215 is necessary for MTs to resist axon retraction induced by contractile actin forces and thereby promotes persistent axon outgrowth [129]. Interestingly, depletion of XMAP215 results in an increased rate of MT plus-end displacement specifically in the growth cone but not in axons. This effect does not seem to depend on XMAP215’s MT-polymerizing function, which relies on plus-end localization, but rather seems to be a result of an additional role for *Xenopus* XMAP215 in MT sliding [129].

The kinesin-4 family member KIF21A is also involved in axon development. In HeLa cells, KIF21A is part of a cortical MT-anchoring complex that includes CLASP, where KIF21A acts as a growth inhibitor to prevent further polymerization of MTs that reach the cell cortex [130]. Missense mutations in KIF21A cause Congenital Fibrosis of the Extraocular Muscles type 1 (CFEOM1), a disease characterized by the patients’ inability to control eye movements due to defects in oculomotor nerve development [131]. These mutations were found to prevent KIF21A autoinhibition, promoting increased cortical MT growth inhibition via an EB-independent interaction of KIF21A with the MT plus-end [130, 132]. Mutant KIF21A results in growth cone and axon pathfinding defects in cultured neurons and knockin mice, suggesting that improper innervation of extraocular muscles in CFEOM1 is a result of misregulation of MT dynamics by KIF21A [130, 132].

## Regulation of interactions between microtubules and +TIPs

It is clear that elaborate control of the MT cytoskeleton, which involves tight regulation of interactions between +TIPs and MTs, is of pivotal importance to neurons.

Since many +TIPs use the same mode of association with EBs, competitive binding between +TIPs from the same subclass is a major factor in the regulation of interactions. This is illustrated by the use of SxIP-motif containing peptides to disrupt +TIP complexes and MT dynamics in the literature (e.g. [133, 134]), and such competition was proposed between CLIP-115 and CLIP-170, for example [85]. However, competition between different subclasses has also been reported. For instance, the SxIP-motif containing Neuron Navigator (NAV) +TIPs have been shown to displace p150glued from MT plus-ends upon overexpression [135], although p150glued relies on CAP-Gly domains to associate with EBs. Small SxIP peptides can also abolish p150glued and CLIP binding to MT plus-ends in in vitro reconstitution assays, confirming that binding sites on EBs for different +TIPs of different subclasses overlap at least partly [133]. Additionally, it will be interesting to see whether +TIPs can provide indirect feedback to other +TIPs by impacting cytoskeleton dynamics or by affecting the conformation of EB proteins.

Not all dominant +TIP-EB interactions negatively impact the recruitment of other +TIPs. For instance, EB1 is sufficient to independently recruit both p150glued and CLIP-170 to the MT plus-end in reconstitution assays using purified proteins, but p150glued binds the plus-end tighter in the presence of CLIP-170 because of an additional interaction between the two +TIPs [133]. A similar mechanism is employed by SLAIN2, which likely uses interactions with multiple +TIPs to overcome the issue of competition [127]. Intuitively, multiplying the number of binding modules should also increase the affinity of +TIPs for the MT plus-end. This has been shown for both the repetition of SxIP motifs within individual proteins and for increases in the number of SxIP motifs via oligomerization [42].

Apart from effects arising from the presence of other +TIPs at the MT plus-end, modifications of MTs or +TIPs provide additional layers of control. At the MT level, post-translational tubulin modifications favor binding of certain +TIPs over others. For example, CAP-Gly +TIPs only associate with MT plus-ends containing tyrosinated α-tubulin [71, 136]. Presence of the C-terminal tyrosine on α-tubulin also promotes MT interaction with another +TIP, the SxIP-containing kinesin-13 KIF2C/MCAK (mitotic centromere-associated kinesin), which has a MT-destabilizing function [137]. MAPs present on the MT lattice may also contribute to regulation of +TIP binding: MAP1B is able to capture cytosolic EBs and immobilize them along MTs, effectively lowering the concentration of EBs at the plus-end of the MT and thereby fine-tuning axon outgrowth [138]. Likewise, MAP2 recruits EBs to the MT lattice in dendrites upon synaptic stimulation [139] and tau was recently reported to bind to EB1. Tau expression levels may also regulate EB localization, as high levels of tau result in EB immobilization along the MT lattice [10].

At the +TIP level, phosphorylation is considered the classic mechanism to regulate binding. Phosphorylation of +TIPs results in unfavorable electrostatic interactions with negatively charged MTs and may promote +TIP accumulation at the MT plus-end rather than along the MT lattice, or abrogate binding altogether [15, 114]. Phosphorylation of +TIPs in the vicinity of SxIP motifs can also suppress binding to the negatively charged C-terminal part of EBs and thus the plus-end tracking [42]. Many +TIPs involved in axon outgrowth are substrates of GSK3β [92, 140–142], a kinase involved in prominent signaling pathways such as the phosphoinositide 3-kinase (PI3K)/Akt pathway and Wnt signaling. In addition to the previously discussed role of CLASP as a GSK3β substrate during axon outgrowth, GSK3β exerts control on the +TIP APC during growth cone advance [140, 143]. Binding of the spectraplakin +TIP MACF1 to MTs is also under control of GSK3β, and the GSK3β-MACF1 interaction plays a role in pyramidal neuron migration [142, 144]. Interestingly, both APC and MACF1 were suggested to regulate GSK3β activity during Wnt signaling [145, 146], hinting at the existence of complex feedback loops between +TIPs and signaling pathways during neurodevelopment.

A second kinase with strong connections to +TIP regulation is the Abelson kinase (Abl). Together with its substrate, Abelson interacting protein (Abi), Abl orchestrates actin dynamics important for *Drosophila* axon guidance and synaptogenesis [90] and appears to link +TIPs to the actin remodeling machinery. The +TIP NAV2 can promote actin polymerization by interacting with Abi at sites targeted by pioneer MTs [147]. Abl also interacts with p140Cap and is required for p140Cap-mediated actin remodeling [148], although this interaction has not yet been explored in neurons. Interestingly, Abl controls axon guidance via CLASP, which is also under control of GSK3β during the same process [90, 93]. Possibly, multiple signaling pathways and kinases act in parallel on the same +TIPs to allow additional levels of control.

Although phosphorylation remains the best-studied mechanism for regulation of MT-+TIP associations, other types of regulation have started to gain attention. For example, EB1 acetylation has been postulated to regulate binding of the SxIP motif-containing +TIP DDA3 during directional cell migration [149]. Intracellular calcium levels determine whether the MACF2 isoform BPAG1n4 localizes to the lattice or to the plus-end of MTs [150]. Finally, since individual EB proteins display different affinities for their binding partners [56, 151], their expression levels can also affect the composition of +TIP networks in manner dependent on the cell type or developmental stage.

## Microtubule plus-end tracking proteins in brain diseases

For certain neurological disorders, MT dynamics have been examined by live imaging of +TIPs. In the case of multiple sclerosis, a neuroinflammatory condition associated with axonal transport defects and motor neuron degeneration, the number of EB3-positive MT plus-ends was found to increase and their directionality was altered in swollen axons of mouse models [152]. MT plus-end dynamics were also investigated in *C. elegans* after axon damage, which can occur after spinal cord injury or stroke in humans. As expected, axon severing generated a large amount of dynamic MT plus-ends at the newly formed tip of the axon, but interestingly axon regrowth depended on the *C. elegans* EB homolog EBP-1 and could be inhibited by overexpression the MT catastrophe-promoting protein EFA-6 (Exchange Factor for Arf6) [153]. Consistently, axon injury or stress induced by the expression of expanded polyglutamine proteins led to increased MT dynamics, which had a neuroprotective role by delaying or counteracting neuron degeneration in *Drosophila* [154].

While such studies strongly imply that regulation of MT dynamics is important in the response to neurological damage and disease, the underlying mechanisms are still poorly understood. Due to their diverse range of neurodevelopmental functions, many +TIPs are involved in human neurological disorders (Table 1), with mutations in certain +TIPs found to directly cause neurological disease. In this section, we highlight two +TIPs and associated diseases for which the role of plus-end tracking has been investigated at least to some extent: p150glued and tau-tubulin kinase 2 (TTBK2). In addition we consider TTBK1, the closest homolog of TTBK2.

### p150glued in Perry syndrome and hereditary motor neuropathy 7B

As described earlier in this review, p150glued is an essential component of the retrograde axonal transport machinery by regulating and positioning dynein at MT plus-ends. Mutations in the CAP-Gly domain of p150glued, which is required for p150glued binding to EBs and MTs [155, 156], cause two distinct neurological disorders: Perry syndrome and hereditary motor neuropathy 7B (HMN7B). HMN7B affects motor neurons. Symptoms ensue in early adulthood and include muscle atrophy, vocal fold paralysis and breathing difficulties [157]. By contrast, Perry syndrome (reviewed in [158]) mainly affects neurons in the substantia nigra but not motor neurons. This rare syndrome manifests itself around 46 years of age and is associated with parkinsonism, depression, hypoventilation and weight loss.

Interestingly, the p150glued mutations that give rise to these different conditions are in close proximity: G59S mutations cause HMN7B [157], and G71(R/E/A), T72P and Q74P mutations were identified in Perry syndrome patients [159]. Differences between these two diseases may in part be explained by the effect of these mutations on the stability of p150glued. In case of the HMN7B mutation, mutant p150glued aggregates and is incorporated into inclusion bodies [160, 161]. However, p150glued’s global folding and stability is largely unaffected by Perry syndrome mutations [161]. This observation is reflected by the presence of dynactin aggregates in motor neurons of HMN7B patients [162], which are believed to contribute to cell death [160]. Such inclusions are less common in Perry syndrome patients [159]. All mutations disturb the interactions between p150glued and EB proteins or MTs [157, 159, 163], but functional differences between mutated forms of p150glued were also reported. The HMN7B mutation globally perturbs axonal transport by disturbing dynactin binding to dynein when mutant p150glued is incorporated in the dynactin complex. Conversely, Perry syndrome mutations do not affect global axonal transport, but G71R p150glued has a dominant negative effect on the initiation of retrograde trafficking from distal axon tips [163]. This function directly depends on p150glued’s interaction with EB proteins at the MT plus-end [76]. Another potentially disease-related mechanism is the MT catastrophe-suppressing function of p150glued, which is disturbed by the Perry syndrome mutation Q74P [77]. p150glued’s ability to suppress catastrophes relies on its binding to MTs and free tubulin dimers, and is independent of EB proteins. p150glued isoforms harboring all domains necessary for these interactions are primarily expressed in neurons, rendering the effect neuron-specific.

p150glued’s involvement in Perry syndrome and HMN7B is an excellent example of how deficiencies in plus-end tracking can result in neurological disease. It underlines the importance of investigating the role of plus-end tracking for +TIPs implicated in disorders of the brain, yet surprisingly, p150glued remains the single best-studied case.

### TTBK1/2 in spinocerebellar ataxia and Alzheimer’s disease

Two other, closely related +TIPs firmly implicated in brain disease are tau-tubulin kinase 1 and 2 (TTBK1 and TTBK2). TTBK1/2 belong to the casein kinase 1 group and share 60 % sequence identity, primarily between their N-terminal kinase domains (reviewed in [164]). Both TTBK proteins contain two SxIP motives in their C-terminal tail [56], which is only present in vertebrates [164].

TTBK2 was the first tau-tubulin kinase to be identified, phosphorylating MAP2 and α-casein in addition to tau and tubulin [165]. TTBK2 is expressed ubiquitously [166] and can phosphorylate tau at sites identified in paired helical filament tau, a hyperphosphorylated tau variant found in the brains of Alzheimer’s disease (AD) patients [166]. Mutations in TTBK2, which yield a mutant protein truncated after the N-terminal kinase domain, cause spinocerebellar ataxia type 11 (SCA11) [167]: a rare neurodegenerative disease of which symptoms include pronunciation difficulties, involuntary eye movement and ataxia [168]. So far, the brain of one SCA11 patient has been examined and revealed the presence of tau deposits among other signs of pathological aging and cerebellar degeneration, raising the possibility that aberrant tau phosphorylation by mutant TTBK2 contributes to SCA11 pathology [167]. In addition, TTBK2 was more recently shown to be required for the formation of cilia by promoting the removal of the centriolar capping protein CEP110 to allow axoneme extension [169]. Diseases resulting from cilia defects, termed ciliopathies, often affect the brain (reviewed in [170]), and mice harboring a mutation that prematurely truncates TTBK2 lack certain neural cell types known to be lost in cilia-depleted animal models [169]. Since the SCA11-mutated form of TTBK2 is unable to initiate ciliogenesis and can interfere with the ciliary function of full-length TTBK2, TTBK2’s function in cilia formation provides a second explanation for the symptoms of SCA11 [169]. However, the EB1-TTBK2 interaction plays no role in the recruitment of TTBK2 to the basal body or in the subsequent initiation of ciliogenesis [171]. Recently, TTBK2 was found to phosphorylate the kinesin-13 family member KIF2A at the plus-end of MTs in an EB-dependent manner. This interaction regulates the binding and thereby depolymerization of MTs by KIF2A in HeLa cells [172]. The same study suggested that TTBK2 can exist in an auto-inhibited conformation, wherein the kinase domain is folded back onto the tail domain containing the SxIP motifs. Binding of EB would liberate the kinase domain and promote kinase activity, presenting an attractive mechanism for how TTBK kinase activity can be regulated at the plus-end of MTs [172]. Interestingly, overexpressed TTBK2 is also able to displace EB1 from the MT plus-end, and TTBK2 affinity to MTs itself appears to be regulated by its autophosphorylation, suggesting that TTBK2 has the potential to regulate +TIP-MT associations [56]. These studies clearly signal the importance of TTBK2’s interaction with EBs and MT plus-ends. The direct relevance of plus-end tracking for neuronal functions of TTBK2 other than ciliogenesis has not been investigated. It is thus currently unclear whether the functions attributed to TTBK2, such as the earlier discussed pathological tau phosphorylation, a role in neuronal migration [172], regulation of the retrieval of synaptotagmin-1 during synaptic vesicle endocytosis [173] or the control of the activity of transport channels including BGT1, a betaine/γ-amino-butyric acid (GABA) transporter [174], require TTBK2 plus-end tracking.

Full-length TTBK1 was only characterized in 2006 as a neuron-specific kinase with the ability to phosphorylate tau on sites associated with AD [175]. During AD, tau becomes hyperphosphorylated, causing it to detach from MTs and form non-soluble aggregates in the cytosol. Certain genetic variations of TTBK1 have been linked to lower risk of developing AD [176, 177], suggesting that TTBK1 and variations in its expression levels may play a role in Alzheimer pathology. Full-length TTBK1 co-localizes with EB1 and bundles MTs at high expression levels independent of its kinase activity [56], but the contribution of TTBK1 plus-end tracking and MT modulation has never been investigated with respect to its function or involvement in disease.

More recently, TTBK1 and TTBK2 were also shown to phosphorylate TDP-43 (transactive response DNA binding protein of 43 kDa) [178], the phosphorylation-driven aggregation of which is a hallmark of amyotrophic lateral sclerosis and frontotemporal lobar degeneration as well as numerous other neurodegenerative diseases [179]. Understanding the cell biology of these kinases both in health and disease, as well as mapping the full range of substrates of TTBKs will be a crucial step towards the identification of targets for therapeutic intervention.

## Future outlook

As versatile regulators of the MT cytoskeleton, +TIPs play important roles in all aspects of neurodevelopment. It is therefore not surprising that +TIPs are involved in many neurological diseases (Table 1). Although more is known about how individual +TIPs affect MT behavior and integrity, in many cases this knowledge is yet to be extrapolated to neuronal MT networks and in some cases insight is lacking altogether.

It is crucial to develop such understanding as pharmacological intervention to inhibit or promote +TIP activity, and thereby affect specific subsets or properties of MTs, likely provides a more elegant and refined approach to therapy development than the use of MT targeting agents that globally impact MT dynamics. The influence of low doses of MT-targeting drugs on neurons has been studied extensively and was found to impact nearly all processes in developing and mature neurons. For example, treatment with 15–75 nM of the MT destabilizer nocodazole impairs the formation (but not outgrowth) of neurites in young dissociated neurons [23]. At 200 nM, nocodazole was also reported to inhibit primary dendrite formation [83] and has the ability to transform mature, mushroom-headed spines of dendrites into filopodia [38]. These morphological spine changes are paired with decreased synaptic response [38]. The MT stabilizing drug taxol, on the other hand, is known to induce the formation of multiple axons in young dissociated neurons at concentrations as low as 3 nM [23], and nanomolar concentrations of both MT stabilizing and destabilizing drugs affect axon pathfinding [28]. The above effects on neurons were mostly found to be a result of altered MT dynamics, rather than drug-specific effects on other cellular processes. It is therefore reasonable to assume that treatment with other MT-targeting agents would result in similar effects on neuronal development and functioning.

MT-targeting agents do provide evidence that drugs altering MT dynamics may be successful in treating brain diseases. For example, the MT stabilizing, blood–brain barrier-penetrant drug epothilone D shows promise as a therapeutic agent in neurological disorders, as epothilone D treatment partially relieves pathology in AD mouse models [180]. Recently, the closely related MT-stabilizing agent epothilone B also proved to stimulate regeneration of axons after spinal cord injury in rats [181]. However, in contrast to the global effect of MT-targeting drugs which can yield unintended side effects, targeting individual +TIPs that are restricted to certain subcellular localizations or tissues, expressed at certain times or influence only limited aspects of MT behavior may be preferable. Even when +TIPs are not involved in establishing pathogenesis, they can potentially be stimulated or inhibited to counteract MT-related disease mechanisms. Of note, it has recently been proposed that the neuroprotective peptide NAP possibly exerts its effects via EBs. NAP affects the brain-specific tubulin pool as well as MT stability and dynamics [182, 183], and has been described to have beneficial effects in tauopathy animal models [184–186]. NAP is believed to bind EB3 via an alternative SxIP motif, Ser-Ile-Pro, and EB silencing in PC12 cells obliterates the protective effect of NAP to zinc toxicity. Although the precise mechanism remains unclear, these data suggest that NAP’s binding to EBs may contribute to its neuroprotective properties [187].

In summary, +TIPs may offer a more precise approach to interfere with pathological MT-related processes, rather than conventional MT-targeting agents currently used in the clinic. In order to truly establish their potential as therapeutic targets for treatment against neurological disorders, more research is needed into the functions and mechanisms of action of an ever-growing pool of +TIPs. New +TIPs are still being recognized on a large scale (e.g. [56]), rendering the field of +TIP research an exciting and promising one for years to come.

## Abbreviations

Abi: Abelson interacting protein
Abl: Abelson kinase
AD: Alzheimer’s disease
AIS: Axon initial segment
BDNF: Brain-derived neurotrophic factor
CAMSAP: Calmodulin-regulated spectrin-associated protein
CAP-Gly: Cytoskeletal-associated protein glycine-rich
CEP: Centrosomal protein
CFEOM1: Congenital fibrosis of the extraocular muscles type 1
CH: Calponin homology
CLASP: Cytoplasmic linker protein-associated protein
CLIP: Cytoplasmic linker protein
DRG: Dorsal root ganglia
EB: End-binding protein
EFA-6: Exchange factor for Arf6
EM: Electron microscopy
ER: Endoplasmic reticulum
GAR: Growth arrest-specific 2 protein-related region
GSK3β: Glycogen synthase kinase 3 beta
HMN7B: Hereditary motor neuropathy 7B
MACF: Microtubule-actin crosslinking factor
MAP: Microtubule-associated protein
MCAK: Mitotic centromere-associated kinesin
MT: Microtubule
NAV: Neuron navigator
NMDA: *N*-Methyl-d-aspartate
PI3K: Phosphoinositide 3-kinase
SCA11: Spinocerebellar ataxia type 11
SOCE: Store operated calcium entry
STIM1: Stromal interaction molecule 1
TDP-43: Transactive response DNA binding protein of 43 kDa
+TIP: Microtubule plus-end tracking protein
TOG: Tumor overexpressed gene
TTBK: Tau-tubulin kinase

## Footnotes

1 With the terminology ‘plus-end out’, we designate MTs with their plus-end oriented towards the cell periphery, or towards dendritic or axonal tips. ‘Minus-end out’ indicates MTs of which the minus-end is oriented towards the cell periphery or process tips.
